# Improved costs and outcomes with conscious sedation vs general anesthesia in TAVR patients: Time to wake up?

**DOI:** 10.1371/journal.pone.0173777

**Published:** 2017-04-05

**Authors:** William Toppen, Daniel Johansen, Sohail Sareh, Josue Fernandez, Nancy Satou, Komal D. Patel, Murray Kwon, William Suh, Olcay Aksoy, Richard J. Shemin, Peyman Benharash

**Affiliations:** 1 UCLA Department of Medicine, Los Angeles, California, United States of America; 2 UCLA Division of Cardiac Surgery, Los Angeles, California, United States of America; 3 UCLA Department of Anesthesia and Preoperative Medicine, Los Angeles, California, United States of America; 4 UCLA Division of Cardiology, Los Angeles, California, United States of America; University of Florida, UNITED STATES

## Abstract

**Background:**

Transcatheter aortic valve replacement (TAVR) has become a commonplace procedure for the treatment of aortic stenosis in higher risk surgical patients. With the high cost and steadily increasing number of patients receiving TAVR, emphasis has been placed on optimizing outcomes as well as resource utilization. Recently, studies have demonstrated the feasibility of conscious sedation in lieu of general anesthesia for TAVR. This study aimed to investigate the clinical as well as cost outcomes associated with conscious sedation in comparison to general anesthesia in TAVR.

**Methods:**

Records for all adult patients undergoing TAVR at our institution between August 2012 and June 2016 were included using our institutional Society of Thoracic Surgeons (STS) and American College of Cardiology (ACC) registries. Cost data was gathered using the BIOME database. Patients were stratified into two groups according to whether they received general anesthesia (GA) or conscious sedation (CS) during the procedure. No-replacement propensity score matching was done using the validated STS predicted risk of mortality (PROM) as a propensity score. Primary outcome measure with survival to discharge and several secondary outcome measures were also included in analysis. According to our institution's data reporting guidelines, all cost data is presented as a percentage of the general anesthesia control group cost.

**Results:**

Of the 231 patients initially identified, 225 (157 GA, 68 CS) were included for analysis. After no-replacement propensity score matching, 196 patients (147 GA, 49 CS) remained. Overall mortality was 1.5% in the matched population with a trend towards lower mortality in the CS group. Conscious sedation was associated with significantly fewer ICU hours (30 vs 96 hours, p = <0.001) and total hospital days (4.9 vs 10.4, p<0.001). Additionally, there was a 28% decrease in direct cost (p<0.001) as well as significant decreases in all individual all cost categories associated with the use of conscious sedation. There was no difference in composite major adverse events between groups. These trends remained on all subsequent subgroup analyses.

**Conclusion:**

Conscious sedation is emerging as a safe and viable option for anesthesia in patients undergoing transcatheter aortic valve replacement. The use of conscious sedation was not only associated with similar rates of adverse events, but also shortened ICU and overall hospital stays. Finally, there were significant decreases in all cost categories when compared to a propensity matched cohort receiving general anesthesia.

## Introduction

Aortic stenosis (AS) is a frequently encountered disease affecting 2–4% of the population over 75 years of age [1]. In recent years, transcatheter aortic valve replacement (TAVR) has steadily grown in popularity as an alternative to traditional surgical intervention for patients with severe AS [2]. While Smith et al [3] showed superiority of TAVR to medical therapy in inoperable patients, it has been demonstrated that TAVR has comparable outcomes to surgical aortic valve replacement (SAVR) in patients with both high and intermediate operative risk (Society of Thoracic Surgeons risk score) [4, 5]. With these expanded indications, application of TAVR is expected to increase in the near future.

Despite its clinical efficacy, a number of investigators have reported TAVR to have considerably higher costs when compared to SAVR. Notably, Reynolds *et al* showed significantly higher cumulative 1-year costs among TAVR patients than patients who received surgical intervention at approximately $60,000/year of life earned [6]. The high resource utilization observed in TAVR may be in part due to the cost of the prosthesis and the prolonged period of recovery from general anesthesia in elderly and frail patients.

Given the success and safety of conscious sedation (CS) in other surgical and interventional procedures [7,8], this anesthetic technique has been recently applied to TAVR. Indeed, TAVR using CS has been associated with less hemodynamic instability and catecholamine use, as well as decreased respiratory and infectious complications [8,9]. The present study was performed to systemically assess the clinical and financial outcomes associated with CS approach to TAVR at our institution.

## Methods

All adult patients undergoing isolated TAVR procedures for symptomatic severe aortic stenosis between August 2012 and June 2016 were identified using our institutional Society of Thoracic Surgeons (STS) database and American College of Cardiology (ACC) Transcatheter Valve Therapy Registry (TVTR). Patients were excluded for missing records, aborted procedures, or significant outliers of cost as described below. Patients were stratified into the general anesthesia (GA) group or conscious sedation (CS) group based on the type of anesthesia delivered at initiation of the procedure. The decision of CS or GA was made on an individual basis by the treatment team consisting of cardiologists, cardiac surgeons and an anesthesiologist.

First-generation Sapien valves (Edwards Lifesciences, Irvine, CA) were initially used in all cases and supplanted gradually in favor of Sapien XT in July 2014, and then by Sapien 3 in July 2015. All patients preferentially underwent transfemoral approach when deemed feasible by preprocedural imaging, otherwise a transapical approach was used, requiring general anesthesia.

Predicted Risk of Mortality (STS PROM) was calculated prior to the procedure by the treatment team using the publicly available STS Risk Calculator. Missing values were supplemented from electronic medical records when necessary and available.

All cost data was collected using our institution’s BIOME Database (BIOME Analytics, Sausalito, CA), a system that is able to merge hospital financial and clinical data. The primary outcome of the study was in-hospital mortality, while secondary outcomes included hospital and ICU lengths of stay, hospital costs, and adverse events. Given the relatively low incidence of major adverse events (MAE’s), a composite variable was developed for analysis consisting of the following MAE’s: all-cause mortality, myocardial infarction (MI), cardiac arrest, new pacemaker requirement, new onset atrial fibrillation, major vascular event, ischemic stroke or TIA, new need for dialysis, gastrointestinal and urinary bleeding(GI/GU), or other bleeding event, annular disruption, hematoma or bleeding at access site, and unplanned or emergent cardiac or vascular surgery. Quality of life as assessed using the validated Kansas City Cardiomyopathy 12-question survey (KCCQ12) [10], was also included as a secondary outcome.

Conscious sedation was defined as “a drug induced depression of consciousness during which patients respond purposefully to verbal commands, either alone or accompanied by light tactile stimulation. No intervention is required to maintain a patent airway, and spontaneous vent is adequate.” General anesthesia was defined as “drug-induced loss of consciousness during which patients are not arousable, even by painful stimulation. The ability to independently maintain ventilator function is often impaired. Patients often require assistance in maintaining a patent airway, and positive pressure ventilation may be required because of depressed spontaneous ventilation or drug-induced depression of neuromuscular function.” [11]

Propensity score matching was used to control for significant intergroup differences and account for potential selection biases. The STS predicted risk of mortality score, a previously validated composite risk stratification, was used as our propensity score [12] (Table 1). All patients without a match were then dropped and all subsequent analyses were completed on the matched cohort.

**Table 1:** Patient Characteristics, all matched patients.

*Clinical Variable*	*Total, n = 196*	*General, n = 147*	*Sedation, n = 49*	*P-value*
*Age, mean (SD), years*	82.7 ± 11	82.4 ± 11	83.5 ± 9	0.45
*Male, No. (%)*	97 (49.5)	74 (50.3)	23 (46.9)	0.68
*BMI, mean (SD), kg/m^2^*	26.9 ± 6	26.8 ± 6	27.3 ± 5	0.59
*NYHA class, No. (%)*				0.09
*Class 1*	19 (9.7)	15 (10.2)	4 (8.2)	
*Class 2*	72 (36.7)	56 (38.1)	16 (32.7)	
*Class 3*	87 (44.4)	59 (40.1)	28 (57.1)	
*Class 4*	18 (9.2)	17 (11.6)	1 (2)	
*STS Risk, Mort % (SD)*	7.5 ± 4	7.6 ± 4	7.2 ± 6	0.65
*Hypertension, No. (%)*	155 (79.1)	117 (79.6)	38 (77.6)	0.77
*Afib/Aflutter, No. (%)*	68 (34.7)	53 (36.1)	15 (30.6)	0.48
*Diabetes mellitus, No. (%)*	65 (33.2)	54 (36.7)	11 (22.5)	0.051
*Prior MI, No. (%)*	70 (35.7)	56 (38.1)	14 (28.6)	0.22
*PAD, No (%)*	31 (15.8)	24 (16.3)	7 (14.3)	0.73
*Hemoglobin, mean% (SD)*	11.7 ± 2.2	11.4 ± 2.2	12.3 ± 12.1	0.02
*Renal Status, No. (%)*				0.68
*Normal (Cr<2.0)*	174 (88.8)	131 (89.1)	43 (87.8)	
*Insufficiency (Cr≥2, not on dialysis)*	14 (7.1)	11 (7.5)	3 (6.1)	
*Failure (dialysis)*	8 (4.1)	5 (3.4)	3 (6.1)	
*Hx stroke or TIA, No. (%)*	31 = 3 (16.8)	25 (17.0)	8 (16.3)	0.91
*LVEF, mean (SD)*	53.6 ± 16	52.9 ± 16	56.7 ± 14	0.24
*KCCQ Score*	47.4 ± 26	46.6 ± 25	49.6 ± 27	0.50
*Approach, No. (%)*				<0.001*
*Transfemoral*	132 (67.4)	83 (56.5)	49 (100)	
*Transapical*	64 (32.7)	64 (43.5)	0 (0)	
*Valve Type, No. (%)*				<0.001*
*Sapien*	57 (29.1)	57 (38.8)	0 (0)	
*Sapien XT*	65 (33.2)	65 (44.2)	0 (0)	
*Sapien 3*	74 (37.8)	25 (17.0)	49 (100)	

BMI, body-mass-index; NYHA, New York Heart Association; STS, Society of Thoracic Surgeons; Mort, mortality risk; Afib/Aflutter, atrial fibrillation or atrial flutter; MI, myocardial infarction; PAD, peripheral vascular disease; TIA, transient ischemic attack; LVEF, left ventricular ejection fraction; KCCQ, Kansas City Cardiomyopathy Questionnaire.

* = statistically significant

**Fig 1:**
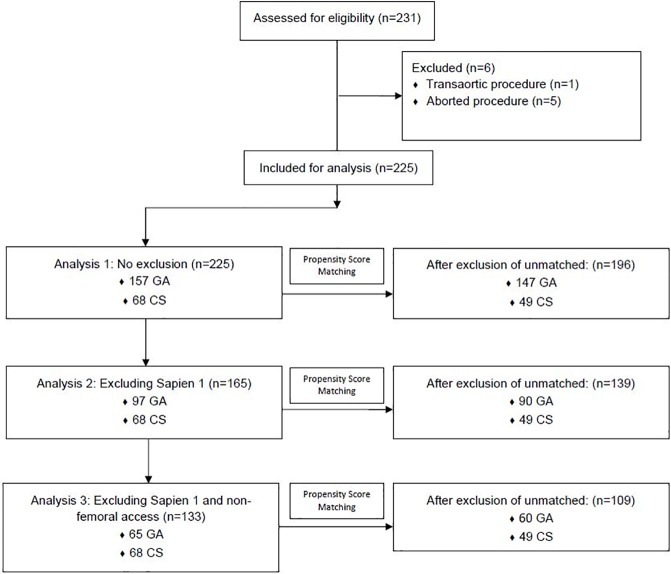
Consort diagram. GA = General Anesthesia group. CS = Conscious Sedation group.

Several distinct analyses were done with different levels of exclusions as illustrated in Fig 1. Firstly, all patients undergoing isolated TAVR procedures with all valve and access choices were analyzed. For the second analysis, all patients with a Sapien 1 (old generation device) were excluded. In the final subanalysis, only patients with newer generation valves (Sapien XT or Sapien 3) and who underwent a transfemoral approach were included in this analysis. The cohorts were propensity matched again after each set of exclusions to assure completeness. (Fig 1).

Continuous values are expressed as the mean and standard deviation and categorical variables as a number and percentage of group. Welch’s t-test of unequal variance and χ^2^- test were used for analysis between groups when appropriate. An alpha of <0.05 was considered statistically significant.

According to institutional policy, actual costs cannot be published. Instead, all cost data is expressed as a percentage of the control (GA group) with a standard deviation. Additionally, cost data used reflects only direct costs, all indirect costs have been omitted.

All statistical analyses were achieved using 13.0 (StataCorp, College Station, TX, USA). This study was approved by the Institutional Review Board at the University of California, Los Angeles.

## Results

### Patient and procedural characteristics

Of the 231 patients identified, 225 (157 GA, 68 CS) met inclusion criteria and underwent further analysis. Five patients were excluded for aborted procedures and one for undergoing a transaortic approach. After no-replacement propensity score matching a total of 196 matched patients (147 GA, 49 CS) remained.

Pre-procedural characteristics were all similar between patient groups with the exception of preoperative hemoglobin (Table 1). Only 1 case was converted from conscious sedation to general anesthesia. Operative characteristic differences between groups persisted after propensity score matching (Table 1) and, as a result, several subsequent subgroup analyses were performed (as detailed below) (Fig 1).

### Clinical outcomes

The overall in-hospital mortality was 1.5% for the matched population with a trend towards a lower mortality in the CS group (0% CS vs 2% GA, *p* = 0.083). Overall, MAE’s occurred with similar frequency in the two groups (33% CS vs 31% GA, *p* = 0.67); however, several individual adverse events were statistically lower in the CS group (Table 2). When compared to GA, patients in the CS group had significantly fewer ICU hours (30±31 vs 96±107, *p* = <0.001) and hospital days (4.9±4 vs 10.4±9.5, *p*<0.001). At one-month follow-up, patients in the CS group had significantly greater improvements in KCCQ12 quality of life scores when compared to GA group (+40 ± 25 vs +29 ± 30, P = 0.025). 30-day readmission rates were no different between groups (Table 2).

**Table 2:** Clinical outcomes, all matched patients.

*Outcomes*	*Total, n = 196*	*General, n = 147*	*Sedation, n = 49*	*P-value*
*Mortality*	3 (1.5%)	3 (2%)	0 (0%)	0.08	
*1+ Major Adverse Event*	69 (35%)	53 (36%)	16 (33%)	0.67	
*MI*	2 (1%)	2 (1.4%)	0 (0%)	0.16	
*Cardiac arrest*	1 (0.5%)	1 (0.7%)	0 (0%)	0.32	
*Major Vasc Event*	2 (1%)	1 (0.7%)	1 (2%)	0.53	
*Ischemic Stroke*	3 (1.5%)	1(0.7%)	2 (4.1%)	0.25	
*TIA*	1 (0.5%)	0 (0%)	1 (2%)	0.32	
*New Dialysis*	7 (3.6%)	7 (4.8%)	0 (0%)	0.008*	
*GI Bleed*	3 (1.5%)	3 (2%)	0 (0%)	0.08	
*GU Bleed*	1 (0.5%)	1 (0.7%)	0 (0%)	0.32	
*Other Bleeding Event*	6 (3.0%)	6 (4.1%)	0 (0%)	0.014*	
*Annular Dissection*	1 (0.5%)	1 (0.7%)	0 (0%)	0.32	
*Hematoma @ site*	3 (1.5%)	2 (1.4%)	1 (2%)	0.76	
*Bleeding @ site*	7 (3.6%)	5(3.4%)	2 (4.1%)	0.83	
*Unplanned surgery*	5 (2.6%)	5 (3.4%)	0 (0%)	0.024*	
*Req Pacemaker*	26 (13%)	18 (12%)	8 (16%)	0.50	
*New onset Afib*	20 (10%)	17 (12%)	3 (6.1%)	0.21	
*ICU Hours*	76 ± 97	96 ± 107	30 ± 31	<0.001*	
*Length of Stay (days)*	9.0 ± 8.8	10.4 ± 9.5	4.9 ± 4	<0.001*	
*30-day readmission*	38 (19%)	30 (20%)	8 (16%)	0.52	
LVEF @ 1 month**	58 ±12	58 ±13	59 ± 12	0.48	
*Change from preop*	+5 ± 11	+5 ±11	+3 ± 10	0.35	
*KCCQ @ 1 month*	81 ± 21	78 ± 22	90 ± 11	<0.001*	
Change from preop***	+32 ± 29	+29 ± 30	+40 ± 25	0.025*	

MI, myocardial infarction; TIA, transient ischemic attack; GI, gastrointestinal; GU, genitourinary; Afib, atrial fibrillation; ICU, intensive care unit; LVEF, left ventricular ejection fraction; KCCQ, Kansas City Cardiomyopathy Questionnaire

* = statistically significant

**LVEF 30 day available for total of 179 patients (135 GA, 44 CS)

***KCCQ 30 day available for total of 172 patients (129 GA, 43 CS)

### Cost outcomes

Cost data was available for all included patients. Costs for the CS group were significantly lower in all categories (Table 3). Direct cost to the hospital for CS was 72% of what it was in GA group (P = <0.001). All other cost categories were less than 50% of GA group.

**Table 3:** Conscious sedation cost outcomes, all matched patients.

*Cost Category*	*% of GA Control Cost*	*P-value*
*Total Direct Cost*	71.5%	<0.001*
*ICU Direct Cost*	45.3%	<0.001*
*Anesthesia Direct Cost*	47.1%	<0.001*
*OR Recovery Direct Cost*	42.6%	<0.001*
*Pharmacy Direct Cost*	42.1%	<0.001*
*Room Direct Cost*	45.5%	<0.001*

ICU, intensive care unit; OR, operating room

* = statistically significant

### Subgroup analysis

Two subgroup analyses were performed as illustrated in Fig 1. The first excluded all Sapien 1 valves and the second excluded all Sapien 1 valves and all non-transfemoral procedures (Tables 4 and 5). After exclusions of Sapien 1 valves, no in-hospital mortalities remained. In both sub-analyses, ICU hours and total length of stay remained significantly less in the CS group (Tables 6 and 7). Additionally, all costs remained significantly lower in the CS group (Tables 8 and 9).

**Table 4:** Patient characteristics; excluding Sapien 1 valves.

*Clinical Variable*	*Total, n = 139*	*General, n = 90*	*Sedation, n = 49*	*P-value*
*Age, mean (SD), years*	82.1 ± 11	81.3 ± 12	83.5 ± 9	0.23
*Male, No. (%)*	72 (52)	46 (51)	26 (53)	0.83
*BMI, mean (SD), kg/m^2^*	27.5 ± 6	27.6 ± 7	27.3 ± 5	0.75
*NYHA class, No. (%)*				0.17
*Class 1*	14 (10.1)	10 (11.1)	4 (8.2)	
*Class 2*	50 (36.0)	34 (37.8)	16 (32.6)	
*Class 3*	65 (46.8)	37 (41.1)	28 (57.1)	
*Class 4*	10 (7.2)	9 (10)	1 (2)	
*STS Risk, Mort % (SD)*	7.2 ± 5	7.2 ± 4	7.2 ± 6	0.95
*Hypertension, No. (%)*	112 (80.6)	74 (82.2)	38 (77.6)	0.52
*Afib/Aflutter, No. (%)*	50 (36.0)	35 (38.9)	15 (30.6)	0.33
*Diabetes mellitus, No. (%)*	45 (32.3)	34 (37.8)	11 (22.4)	0.06
*Prior MI, No. (%)*	52 (37.4)	38 (42.2)	14 (28.6)	0.11
*PAD, No (%)*	19 (13.4)	12 (13.3)	7 (14.3)	0.89
*Hemoglobin, mean% (SD)*	11.8 ± 2.4	11.6 ± 2.4	12.3 ± 2.1	0.07
*Renal Status, No. (%)*				0.50
*Normal (Cr<2.0)*	125 (89.9)	82 (91.1)	43 (87.8)	
*Insufficiency (Cr≥2, not on dialysis)*	9 (6.5)	6 (6.7)	3 (6.1)	
*Failure (dialysis)*	5 (3.6)	2 (2.2)	3 (6.1)	
*Hx stroke or TIA, No. (%)*	24 (17.3)	16 (17.8)	8 (16.3)	0.83
*LVEF, mean (SD)*	54.1 ± 16	53.2 ± 17	55.7 ± 14	0.35
*KCCQ Score*	49 ± 26	49 ± 26	50 ± 27	0.85
*Approach, No. (%)*				<0.001*
*Transfemoral*	109 (78.4)	60 (66.7)	49 (100)	
*Transapical*	30 (21.6)	30 (33.3)	0 (0)	
*Valve Type, No. (%)*				<0.001*
*Sapien XT*	65 (46.8)	65 (44.2)	0 (0)	
*Sapien 3*	74 (53.2)	25 (17.0)	49 (100)	

BMI, body-mass-index; NYHA, New York Heart Association; STS, Society of Thoracic Surgeons; Mort, mortality risk; Afib/Aflutter, atrial fibrillation or atrial flutter; MI, myocardial infarction; PAD, peripheral vascular disease; TIA, transient ischemic attack; LVEF, left ventricular ejection fraction; KCCQ, Kansas City Cardiomyopathy Questionnaire

* = statistically significant

**Table 5:** Patient characteristics; excluding Sapien 1 valves and transapical approach.

*Clinical Variable*	*Total, n = 109*	*General, n = 60*	*Sedation, n = 49*	*P-value*
*Age, mean (SD), years*	81.8 ± 12	80.3 ± 14	83.5 ± 9	0.15
*Male, No. (%)*	53 (51%)	27 (45%)	26 (53%)	0.41
*BMI, mean (SD), kg/m^2^*	27.9 ± 7	28.5 ± 8	27.3 ± 5	0.33
*NYHA class, No. (%)*				0.23
*Class 1*	11 (10%)	7 (12%)	4 (8.2%)	
*Class 2*	40 (37%)	24 (40%)	16 (33%)	
*Class 3*	52 (48%)	24 (40%)	28 (57%)	
*Class 4*	6 (5.5%)	5 (8.3%)	1 (2.0%)	
*STS Risk, Mort % (SD)*	7.1 ± 6	7.0 ± 4	7.2 ± 6	0.79
*Hypertension, No. (%)*	84 (77%)	46 (77%)	38 (78%)	0.91
*Afib/Aflutter, No. (%)*	35 (32%)	20 (33%)	15 (31%)	0.76
*Diabetes mellitus, No. (%)*	35 (32%)	24 (40%)	11 (22%)	0.048*
*Prior MI, No. (%)*	38 (35%)	24 (40%)	14 (29%)	0.21
*PAD, No (%)*	9 (8.3%)	2 (3.3%)	7 (14%)	0.053
*Hemoglobin, mean% (SD)*	11.9 ± 2.4	11.6 ± 2.6	12.3 ± 2.1	0.12
*Renal Status, No. (%)*				0.79
*Normal (Cr<2.0)*	97 (89%)	54 (90%)	43 (88%)	
*Insufficiency (Cr≥2, not on dialysis)*	7 (6.4%)	4 (6.7%)	3 (6.1%)	
*Failure (dialysis)*	5 (4.6%)	2 (3.3%)	3 (6.1%)	
*Hx stroke or TIA, No. (%)*	19 (17%)	11 (18%)	8 (16%)	0.79
*LVEF, mean (SD)*	54.7 ± 16	53.9 ± 17	55.6 ± 14	0.56
*KCCQ Score*	48.9 ± 26	48.2 ± 26	49.6 ± 27	0.79
*Valve Type, No. (%)*				<0.001*
*Sapien XT*	43 (46.8%)	43 (72%)	0 (0%)	
*Sapien 3*	66 (53.2%)	17 (28%)	49 (100%)	

BMI, body-mass-index; NYHA, New York Heart Association; STS, Society of Thoracic Surgeons; Mort, mortality risk; Afib/Aflutter, atrial fibrillation or atrial flutter; MI, myocardial infarction; PAD, peripheral vascular disease; TIA, transient ischemic attack; LVEF, left ventricular ejection fraction; KCCQ, Kansas City Cardiomyopathy Questionnaire

* = statistically significant

**Table 6:** Clinical outcomes, excluding Sapien 1 valves.

*Outcomes*	*Total, n = 139*	*General, n = 90*	*Sedation, n = 49*	*P-value*
*Mortality*	0 (0%)	0 (0%)	0 (0%)	
*1+ Major Adverse Event*	49 (35%)	33 (37%)	16 (33%)	0.64
*MI*	0 (0%)	0 (0%)	0 (0%)	NA
*Cardiac arrest*	1 (1%)	1 (1%)	0 (0%)	0.32
*Major Vasc Event*	1 (1%)	0 (0%)	1 (2%)	0.32
*Ischemic Stroke*	3 (2%)	1(1%)	1 (4%)	0.34
*TIA*	1 (1%)	0 (0%)	1 (2%)	0.32
*New Dialysis*	3 (2%)	3 (3%)	0 (0%)	0.08
*GI Bleed*	1 (1%)	1 (1%)	0 (0%)	0.32
*GU Bleed*	0 (0%)	0 (0%)	0 (0%)	NA
*Other Bleeding Event*	2 (1.4%)	2 (2%)	0 (0%)	0.16
*Annular Dissection*	1 (1%)	1 (1%)	0 (0%)	0.32
*Hematoma @ site*	2 (1.4%)	1 (1%)	1 (2%)	0.69
*Bleeding @ site*	6 (4.3%)	4 (4.4%)	2 (4.1%)	0.92
*Unplanned surgery*	2 (1.4%)	2 (2%)	0 (0%)	0.16
*Req Pacemaker*	24 (17.3%)	16 (17.8%)	8 (16.3%)	0.83
*New onset afib*	16 (11.5%)	13 (14.4%)	3 (6.1%)	0.10
*ICU Hours*	61 ± 67	78 ± 74	30 ± 31	<0.001*
*Length of Stay (days)*	8.1 ± 8.7	9.8 ± 10.1	4.9 ± 4.0	<0.001*
*30-day readmission*	26 (18.7%)	18 (20%)	8 (16.33%)	0.59
LVEF @ 1 month**	59 ±13	58 ±13	59 ± 12	0.74
*Change from preop*	+5 ± 11	+5 ±11	+3 ± 10	0.33
KCCQ @ 1 month***	83 ± 19	79 ± 22	90 ± 11	<0.001*
*Change from preop*	+33 ± 28	+30 ± 29	+40 ± 25	0.037*

MI, myocardial infarction; TIA, transient ischemic attack; GI, gastrointestinal; GU, genitourinary; Afib, atrial fibrillation; ICU, intensive care unit; LVEF, left ventricular ejection fraction; KCCQ, Kansas City Cardiomyopathy Questionnaire

* = statistically significant

**LVEF @ 1 month available for 130 patients (86 GA, 44 CS)

***KCCQ @ 1 month available for 126 patients (83 G, 43 CS)

**Table 7:** Clinical outcomes, excluding Sapien 1 valves and transapical approach.

*Outcomes*	*Total, n = 109*	*General, n = 60*	*Sedation, n = 49*	*P-value*
*Mortality*	0 (0%)	0 (0%)	0 (0%)	
*1+ Major Adverse Event*	36 (33%)	20 (33%)	16 (33%)	0.94
*MI*	0 (0%)	0 (0%)	0 (0%)	NA
*Cardiac arrest*	0 (0%)	0 (0%)	0 (0%)	NA
*Major Vasc Event*	1 (1%)	0 (0%)	1 (2%)	0.32
*Ischemic Stroke*	2 (2%)	0 (0%)	2 (4%)	0.16
*TIA*	1 (1%)	0 (0%)	1 (2%)	0.32
*New Dialysis*	2 (2%)	2 (3%)	0 (0%)	0.16
*GI Bleed*	0 (0%)	0 (0%)	0 (0%)	NA
*GU Bleed*	0 (0%)	0 (0%)	0 (0%)	NA
*Other Bleeding Event*	1 (1%)	1 (2%)	0 (0%)	0.32
*Annular Dissection*	0 (0%)	0 (0%)	0 (0%)	NA
*Hematoma @ site*	2 (2%)	1 (2%)	1 (2%)	0.89
*Bleeding @ site*	6 (6%)	4 (7%)	2 (4%)	0.55
*Unplanned surgery*	2 (2%)	2 (3%)	0 (0%)	0.16
*Req Pacemaker*	18 (17%)	10 (17%)	8 (16%)	0.96
*New onset afib*	11 (10%)	8 (13%)	3 (6%)	0.20
*ICU Hours*	47 ± 44	61 ± 48	30 ± 31	<0.001*
*Length of Stay (days)*	7.1 ± 8.0	8.8 ± 9.8	4.9 ± 4.0	0.006*
*30-day readmit*	20 (18%)	12 (20%)	8 (16%)	0.62
LVEF @ 1 month**	59 ±12	59 ± 13	57 ± 12	0.96
*Change from preop*	+5 ± 10	+6 ± 10	+3 ± 10	0.26
KCCQ @ 1 month***	88 ±15	86 ± 18	90 ± 11	0.11
*Change from preop*	+38 ± 26	+37 ± 27	+40 ± 25	0.47

MI, myocardial infarction; TIA, transient ischemic attack; GI, gastrointestinal; GU, genitourinary; Afib, atrial fibrillation; ICU, intensive care unit; LVEF, left ventricular ejection fraction; KCCQ, Kansas City Cardiomyopathy Questionnaire

* = statistically significant

**30 day LVEF available for 102 patients (58 GA, 44 CS)

***30 day KCCQ scores available for 98 patients (55 GA, 43 CS)

**Table 8:** Conscious sedation cost outcomes, matched patients excluding Sapien 1 valves.

*Cost Category*	*% of GA Control Cost*	*P-value*
*Total Direct Cost*	73.8%	<0.001*
*ICU Direct Cost*	47.0%	<0.001*
*Anesthesia Direct Cost*	57.8%	<0.001*
*OR Recovery Direct Cost*	44.7%	<0.001*
*Pharmacy Direct Cost*	45.8%	0.001*
*Room Direct Cost*	46.7%	<0.001*

ICU, intensive care unit; OR, operating room

* = statistically significant

**Table 9:** Conscious sedation cost outcomes, matched patients excluding Sapien 1 valves and transapical approach.

*Outcomes*	*% of GA Control cost*	*P-value*
*Total Direct Cost*	74.9%	<0.001*
*ICU Direct Cost*	51.2%	0.002*
*Anesthesia Direct Cost*	60.3%	<0.001*
*OR Recovery Direct Cost*	47.8%	<0.001*
*Pharmacy Direct Cost*	48.3%	0.034*
*Room Direct Cost*	52.8%	0.005*

ICU, intensive care unit; OR, operating room

* = statistically significant

## Discussion

Recent introduction of lower profile delivery systems and a wider range of valve sizes has allowed more patients to receive TAVR through the transfemoral approach. During the natural evolution of TAVR technology, fast-track protocols and other procedural modifications have been studied to reduce the cost of this procedure and improve clinical outcomes [8]. Nonetheless, the heavy resource utilization of TAVR technology remains a major concern.

In this study of TAVR patients with high operative risk, we found that the use of conscious sedation was associated with improved hospital and ICU length of stay, improved quality of life measures, and decreases in nearly every cost category. In our analysis of newer generation TAVR prostheses deployed via the transfemoral approach, patients who received CS had at least 30 fewer ICU hours and 4 fewer total hospital days as well as 25% reduced cost when compared to GA.

Others have previously evaluated protocols for improving clinical outcomes in the frail TAVR population. In a feasibility study, Jensen et al reported acceptable outcomes with a “minimalist approach” to TAVR, a cornerstone of which was CS [13]. Similarly, Frohlich and colleagues reported comparable clinical outcomes with CS when compared to GA [8]. Interestingly, our study found improvements in clinical outcomes with CS among TAVR patients. It is plausible that CS leads to less hemodynamic instability, and need for vasoactive agents which may allow lower acuity care and “fast-tracking.” Additionally, less time in the hospital and ICU hours in particular, has been independently shown to decrease risk of nosocomial infections and overall mortality [14].

Our reporting of cost outcomes associated with the institution of CS in the TAVR population highlights several points. With an average 25% reduction in direct costs, reduced ICU and room utilization accounted for the largest reductions with the introduction of CS. However, operating room, pharmacy, and all directs costs were also lower with this method. We performed several sublevel analyses controlling for the type of valve and delivery systems. Even in the most restrictive analysis including only Sapien XT and Sapien 3 valves deployed via the transfemoral approach, we found reductions in nearly all cost categories associated with CS. With an average cost of $55K for TAVR reported by Babaliaros [15], this reduction in costs would translate to nearly $14K per case. While institutional costs may vary significantly, this study provides encouraging financial outcomes for administration of CS in TAVR patients.

Finally, among the frail TAVR population, quality of life is an important consideration. We used the validated KCCQ12 questionnaire to evaluate patient quality of life prior to their procedure and at their 30-day follow-up. Patients who underwent CS TAVR were found to have higher quality of life scores at 30-days in two of three analyses. This may be explained by stress (hemodynamic instability, increased postprocedural interventions, etc) on the body allowing more robust response to treatment or possibly shorter hospital stays leading to more favorable experiences with patients.

## Limitations

This was a single-center, retrospective review of prospectively maintained databases and, as such, has certain limitations. Given the novelty of TAVR as a procedure, our study was limited to a four-year span and relatively small sample size. Additionally, as stated previously, our institution prohibits publication of actual cost data, requiring that we publish only percent change. Finally, as this study encompassed only a four-year span and found a significant difference in cost outcomes, we did not account for inflation.

## Conclusion

Conscious sedation is a safe and viable anesthetic option in patients undergoing transfemoral transcatheter aortic valve replacement. In addition to shorter hospital stays, there is a significant reduction in nearly all cost categories with the use of conscious sedation. Whenever possible, eligible patients undergoing TAVR procedures should preferentially receive conscious sedation in lieu of general anesthesia.
